# New insights into the cellular activities of Fndc5/Irisin and its signaling pathways

**DOI:** 10.1186/s13578-020-00413-3

**Published:** 2020-03-30

**Authors:** Farzaneh Rabiee, Liana Lachinani, Sarvenaz Ghaedi, Mohammad Hossein Nasr-Esfahani, Timothy L. Megraw, Kamran Ghaedi

**Affiliations:** 1 Department of Cell and Molecular Biology and Microbiology, Faculty of Biological Science and Technology, University of Isfahan, Isfahan, Iran; 2 Department of Cellular Biotechnology, Cell Science Research Center, Royan Institute for Biotechnology, ACECR, Royan St., Salman St, 816513-1378 Isfahan, Khorsagan Iran; 3 Department of Molecular Biotechnology, Cell Science Research Center, Royan Institute for Biotechnology, ACECR, Isfahan, Iran; 4 Department of Biomedical Sciences, Florida State University College of Medicine, West Call Street, Tallahassee, FL 32306-4300 USA

## Abstract

Fndc5, a well-defined myokine and also identified as an adipokine, has a critical role in modulation of metabolism and protection against obesity. These important functions are mediated by irisin, a secretory peptide produced from proteolytic processing of Fndc5. The other beneficial physiological effects of irisin are alleviation of oxidative stress, neuroprotective effects, and anti-inflammatory properties and associated anti-metastatic effects. Fndc5/irisin exerts its biological effects through several intracellular signaling pathways. The major signaling pathway is thought to be MAPK signaling pathways which are involved in neural differentiation, browning of white adipocytes, as well as osteoblast proliferation and differentiation. Other essential functions of Fndc5/irisin are mediated through additional pathways including AMPK pathway, PI3K/AKT, and STAT3/Snail. Thorough understanding of the mechanisms of irisin actions are essential in order to develop Fndc5/irisin for therapeutic purposes. In the present review, we focus on the current knowledge of the signaling pathways that elicit irisin actions.

## Background

Fibronectin type III domain-containing protein 5 (FNDC5), also called fibronectin type III repeat containing protein (FRCP2) and Peroxisomal Protein (Pep) was first discovered and characterized in 2002 by two independent groups [1, 2]. Böstrom and colleagues first reported increased *FNDC5* transcript levels in the skeletal muscle of mice and humans after exercise. *FNDC5* encodes a PGC1α-dependent myokine, as a part Fndc5 protein is proteolytically processed and secreted as irisin, which can promote conversion of white adipose tissue (WAT) to brown adipose tissue (BAT) by increased *UCP1* expression [3]. *Fndc5* transcript is expressed in multiple tissues including the heart, brain, ovary, testis, kidney, stomach and liver [4]. Literature mining indicates that Fndc5 not only plays a vital role in energy metabolism but also it has crucial roles in a variety of processes such as inflammation, proliferation, metastasis and neural differentiation. In this review we cover the best-understood cellular signaling pathways that Fndc5/irisin acts to elicit these physiological effects.

### Fndc5 and MAPK signaling pathways

**Fig. 1:**
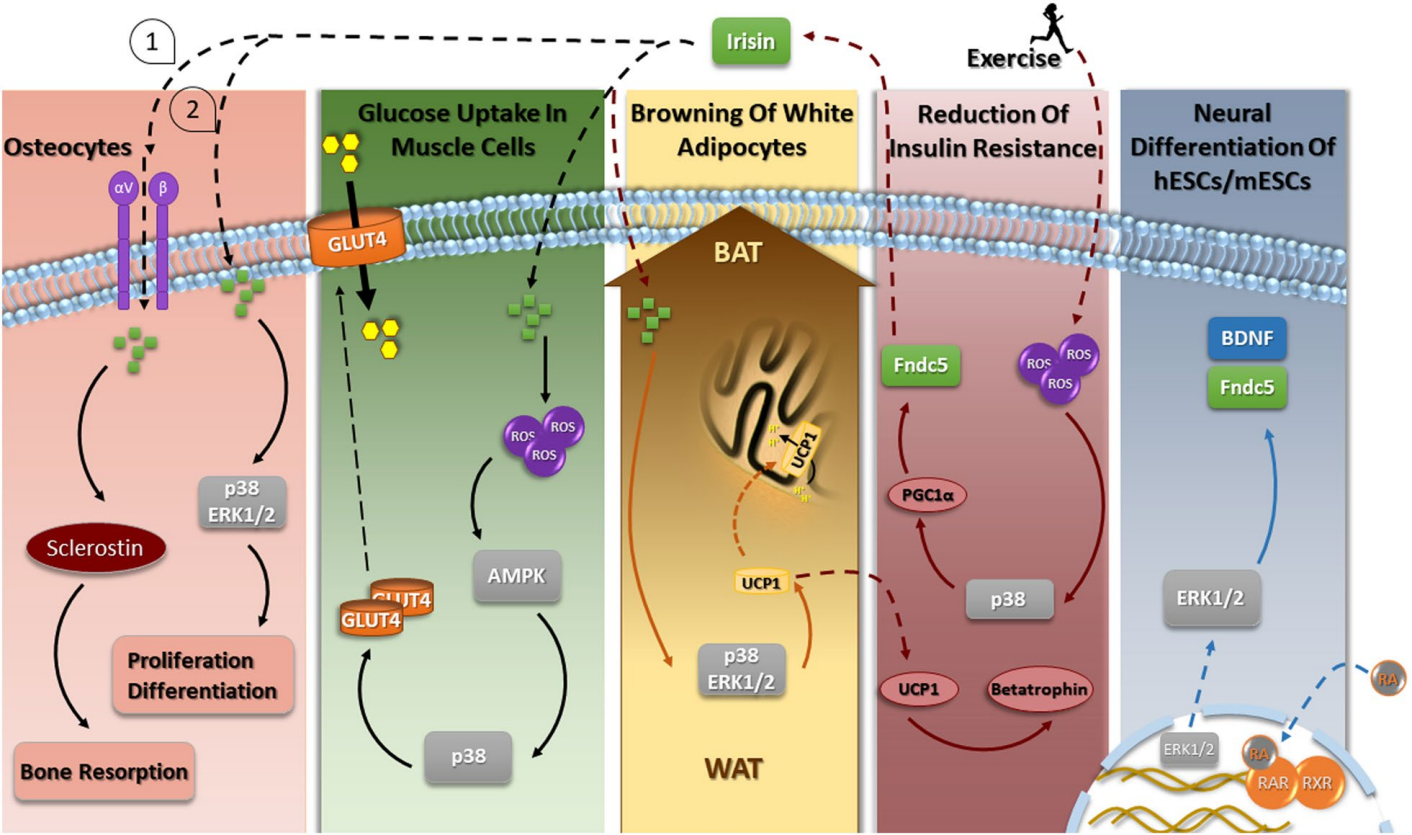
Schematic representation of the the main physiological activities mediated by Fndc5/Irisin through MAP-kinase signaling pathways. The variety of cell differentiation and physiological activities of Fndc5/irisin and the MAPK pathways they elicit are depicted. As shown, through this signaling pathway, irisin is not only responsible for neural cells and osteocytes differentiation but also triggers glucose uptake by the muscles and browning of WAT

The mitogen-activated protein kinases (MAPKs) regulate a variety of cellular processes by relaying extracellular signals to intracellular responses [5]. MAPK signaling impacts multiple fundamental cellular processes such as gene expression, mitosis, metabolism, motility, survival, apoptosis, and differentiation. The best understood MAPKs are the conventional MAPKs: The c-Jun N-terminal kinases 1–3 (JNK1-3), extracellular signal-regulated kinase 1 and 2 (ERK1/2), the p38 isoforms (α, β, γ, and δ) and ERK5 families. Among these, the less-understood MAPKs are Erk3/4, and Erk7/8 and stress activated protein kinases (SAPK1A, 1B, 1C) [5, 6]. The extracellular stimuli include environmental stressors, growth factors, and cytokines, which activate MAPKs via both receptor-dependent and -independent mechanisms. Each group of conventional MAPKs is composed of a set of three evolutionarily conserved, sequentially acting kinases: a MAPK, a mitogen-activated protein kinase kinase (MAPKK), and a mitogen-activated protein kinase kinase kinase (MAPKKK) [5]. The major functions regulated by the MAPKs are mediated through their phosphorylation of a variety of protein substrates; including members of a family of protein kinases termed MAPK activated protein kinases (MAPKAPKs). Recent studies indicate that Fndc5 acts mostly through MAPK signaling pathways in numerous cellular processes (Fig. 1). A list of physiological effects of irisin and downstream pathways are shown in Table 1.

**Table 1:** Diverse Fndc5/Irisin functions and the associated signaling pathways

Source of irisin/Fndc5	Target cells/tissue	Signaling pathway	Effects	References
Mouse embryoid bodies	Mouse Embryoid Bodies	ERK1/2 MAPK pathway	Facilitates neural differentiation	[8]
Recombinant irisin	White Adipocytes	p38 and ERK MAP Kinase	Browning of white adipocytes	[21]
Conditioned medium of 3T3-L1 cell line and myoblast	Primary rat osteoblast and MC3T3-E1 cell line and bone marrow stromal cells	p38 and ERK MAP Kinase	Osteoblast proliferation and differentiation	[27–29]
Recombinant irisin	Human endothelial cell	ERK1/2 MAPK	Endothelial cell proliferation	[34]
Recombinant irisin	Primary-cultured myoblasts and L6 cells	P38 MAPK	Glucose uptake	[35]
circulating irisin	Skeletal muscle	p38 MAPK and ERK MAPK	Insulin sensitivity	[40]
Recombinant irisin	H19-7 hippocampal cell lines	STAT3	H19-7 cell proliferation	[47]
Fndc5 overexpression circulating irisin	Adipose tissue	AMPK pathway	Attenuates inflammation	[52]
Recombinant irisin	A549 and NCI-H446 lung cancer cells	PI3K/AKT pathway	Anti-metastatic effects	[56]
Recombinant irisin	U2OS cells (osteosarcoma cells)	STAT3/Snail signaling pathway	Anti-metastatic effects	[62]
Skeletal muscle	White adipose tissue	AMPK-PGC1α-FNDC5 signaling pathway	Browning of WAT	[65]
Recombinant irisin	RAW-264.7 cell line	TLR4/MyD88 Signaling Pathway	Anti-Inflammatory	[66]
Recombinant irisin	Endothelial cells	AMPK-Akt-eNOS-NO Pathway	Lowers Blood Pressure	[68]
Recombinant irisin	Cardiomyocyte and mouse heart	AMPK-ULK1 and AMPK- mTOR	Improves cardiac hypertrophy	[82, 83]
Recombinant irisin	Human cortical slices Mouse hippocampal slices	cAMP/PKA/CREB	Roles in memory formation	[46]
Recombinant irisin	PaCa-2 and Panc03.27 cells	AMPK-mTOR	Suppress pancreatic cancer cell	[63]
Recombinant irisin	3T3-L1	Wnt signaling	Inhibit adipogenesis	[42]
Hippocampus of mice	Hippocampus of mice	FNDC5/BDNF/Akt	antidepressant-like effect	[81]

### Fndc5 plays a vital role in neural differentiation through ERK1/2 pathway

The vital role of Fndc5 in the process of neural differentiation and protection have been shown in many studies [7–15]. *Fndc5* expression elevates after retinoic acid (RA) treatment of mouse embryonic stem cells (mESCs) in the process of neural differentiation [7]. The importance of *Fndc5* in neural differentiation process was shown by loss and gain of function studies [9, 10]. RA binds to its nuclear receptor, retinoic acid receptor (RAR), and then acts as a transcription factor to affect RA-responsive genes, including induction of the genes encoding MAPKs (ERK1/2, JNK, P38) [16–19]. RA treatment of mouse embryoid bodies (EBs) elevated ERK1/2 activity, triggering an increase in *Fndc5* and *BDNF* transcript levels in neural differentiation of mouse and human embryonic stem cells. Consistently, ERK1/2 loss of function significantly decreased *Fndc5* and *BDNF* expression during neural differentiation [8] (Table 1).

### Browning of white adipocytes is mediated by Fndc5 through p38 and ERK MAP kinase signaling

In its critical role regulating energy metabolism, irisin exerts beneficial effects through the conversion of WAT to BAT that is associated with weight loss and improved glucose homeostasis [3]. Owing to this important physiological regulation, irisin shows great therapeutic potential in diabetes and obesity [20]. Irisin induces the browning of WAT through p38 and ERK signaling [21]. Phosphorylated p38 (P-p38) and phosphorylated ERK (P-ERK) were both significantly increased following treatment with recombinant irisin in both primary rat and 3T3-L1 adipocytes. Irisin up-regulated uncoupling protein-1 (UCP1) in this process, a response that was blocked with drugs that inhibit p38 or ERK [21] (Table 1).

### Irisin triggers osteoblast proliferation and differentiation via p38 and ERK signaling pathways

Bone metabolic diseases are a diverse group of bone metabolism disorders, mostly characterized by decreased bone mineral density in calcium or phosphorous and vitamin D [22, 23]. These diseases have a significant impact on the elderly population [23]. Exercise, by maintaining bone mass and strength, prevents bone cell diminishment and acts as a vital anti-aging factor for preserving bone integrity [24–26]. Several studies recently have shown that irisin acts as an exercise induced hormone, which promotes osteoblast proliferation and differentiation through activating the p38 and ERK [27–29]. Involvement of these signaling pathways in osteogenesis was confirmed by inhibition of each signaling pathways, using their inhibitors [28]. On the other hand, bone loss was prevented and osteoporosis was blocked in mice lacking irisin. Kim et al. showed that irisin binds directly to α_v_ integrin receptors on osteocyte cells and induces the expression of sclerostin which involves bone resorption by increasing osteoclasts activity [30]. Therefore, the therapeutic potential of irisin in bone metabolism disease is unclear but has strong potential (Table 1).

### p38–PGC-1α–irisin–betatrophin axis decreases insulin resistance

A new hormone, betatrophin, has recently been identified that contributes to pancreatic β-cell regeneration and specifically increases β-cell mass in mice. Betatrophin is connected by a new pathway involved in insulin resistance [31]. Irisin basically acts on WAT cells after endurance exercise training through induction of *UCP1* expression and energy expenditure [3]. Irisin not only induces *UCP1* expression through MAPK pathways (P38 and ERK) but also promotes the expression of betatrophin through these pathways [31] (Table 1).

### Fndc5 regulates endothelial cell proliferation through the ERK1/2 MAPK pathway

The integrity of endothelial cells is critical, as their dysfunction is responsible for a variety of vascular diseases like chronic metabolic disease [32]. Endothelial cell proliferation is vital for new blood vessel growth during angiogenesis, especially in diseases like type II diabetes to support wound healing [33]. In addition to its role in regulating metabolic homeostasis, irisin may stimulate proliferation in some cell types. Irisin increases human umbilical vein endothelial cell (HUVEC) proliferation by activating ERK signaling pathways, potentially supporting new blood vessel growth. Irisin also promotes INS-1 cell proliferation via the ERK and p38 MAPK signaling pathways [34] (Table 1).

### Glucose uptake and homeostasis of Fndc5 through P38 MAPK and ERK pathways

Skeletal muscle is the main source of irisin production. Irisin has diverse physiological functions including thermogenesis, glucose metabolism, increased metabolism, differentiation, and proliferation [3]. A recent study showed that irisin stimulates glucose uptake in muscle cells through p38 signaling activated by ROS-mediated AMPK activation [35]. β-arrestin-2 also has an active role in irisin induced glucose metabolism in type 2 diabetes mellitus (T2DM) by controlling the p38 MAPK signaling. Irisin not only elevates glucose uptake but also plays an essential role in glucose homeostasis by direct effects on adipose tissue, muscle, liver and pancreas and sometimes by indirect effects through synergistic effects with other hormones. In adipose tissue in addition to increase in *UCP1* expression via p38 MAPK and ERK pathways, glucose homeostasis, is mediated by lipolysis stimulation through the cyclic AMP–protein kinase A (PKA)–perilipin–hormone-sensitive lipase (HSL) pathway [36]. These findings present a novel therapeutic avenue for potential treatment of diabetes [37] (Table 1).

### Relevance between irisin and insulin signaling through p38 and ERK MAPKs

A positive association between irisin and insulin resistance is reported in muscle [4, 38, 39]. Palmitate treatment of the C2C12 cell line resulted in decreased AKT and ERK signaling and irisin antagonized the reduced or diminished phosphorylation of AKT and ERK after palmitate treatment [40]. To understand the fundamental role of irisin in the amelioration of insulin resistance in involved tissues especially muscle tissue, more experiments are needed (Table 1).

Taken together, it seems that MAPK signaling pathways which are involved in cellular energy expenditure, cell proliferation and differentiation in a variety of cell and tissue types could be activated by irisin. This feature of irisin function is a very critical aspect physiologically for coordination between metabolic rate of tissues and organs which is governed by muscle and fat tissues as the main secretory sources of irisin.

### Other signaling pathways for Fndc5/irisin function

**Fig. 2:**
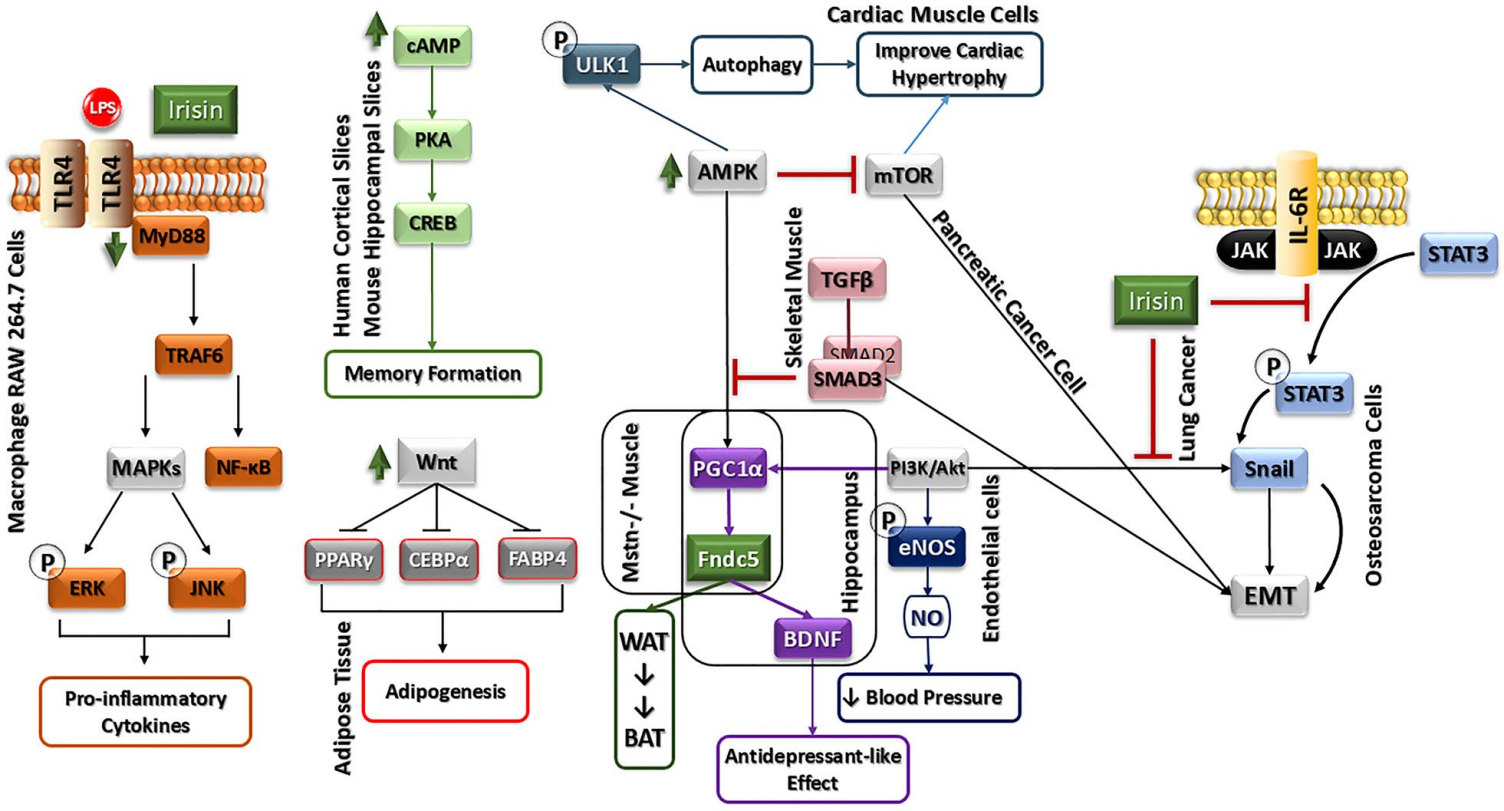
Graphic summary of physiological activities of Fndc5/Irisin that are elicited through pathways other than MAP-kinase signaling. Alternative signaling pathways that transmit the effects of Fndc5/Irisin are AMP-kinase, STAT3 and the TLR4 pathways. These pathways are involved in a set of cellular functions responsible for proliferation, anti-metastatic and anti-inflammatory activities. Irisin improves cardiac hypertrophy by inducing protective autophagy via mTOR independent activation of AMPK-ULK1 and AMPK- mTOR pathways. On the other hand, Irisin has an antidepressant-like effect in hippocampus. Of note that, Irisin lowers blood pressure via the AMPK-Akt-eNOS-NO pathway in endothelial cells. Anti-inflammatory properties of irisin are connected to TLR4/MyD88 signaling pathway activation in macrophages. For detailed information please see the text

Fndc5/irisin could act through alternative signaling pathways. The outline of these pathways is shown in Fig. 2 as follows:

### Adipogenesis is suppressed by irisin through Wnt signaling

In addition to myokine activity, Irisin can also act as type of adipokine. Therefore, besides of the main role of irisin which is browning of WAT, irisin also prohibits accumulation of lipids through up-regulation of adipose triglyceride lipase (ATGL) and down regulation of fatty acid synthase (FAS) [41]. This modulatory effect is mediated by the PPARγ, C/EBPa, and FABP4 axis under control of Wnt signaling, as FNDC5/irisin up regulates Wnt6 and Wnt10a and Wnt 10b [42]. Wnt signaling is inhibitory for adipocyte differentiation [43]. This aspect of Irisin function through Wnt signaling is complementary to browning of WAT which is mediated by MAPK signaling pathways to suppress of body fat percentage (BFP) in the human body. Therefore, it can be concluded that the main aspect of irisin secretion through the muscle is governing of fat size of the body and thereby governing the metabolic rate of fat tissue.

### Irisin has a neuroprotective role through stimulation of cAMP/PKA/CREB pathway

The role of FNDC5/irisin in learning and memory is mediated through the expression of brain-derived neurotrophic factor (BDNF) in the hippocampus [44]. This finding supports the potential role for FNDC5/irisin in preventing brain disorders such as Alzheimer’s disease (AD).

CREB (cAMP response element-binding protein) is a cellular transcription factor which has a well-documented role in neuronal plasticity and long-term memory formation in the brain [45]. Recent studies have revealed that recombinant irisin stimulates the cAMP/PKA/CREB pathway not only in humane cortical slices but also in mouse hippocampal slices [46].

### Irisin increases H19-7 cell proliferation by activating STAT3 pathway

Irisin is expressed in the human brain [4, 13]. We have also shown that knockdown of Fndc5 decreased neural differentiation of mouse embryonic stem cells whereas its overexpression increased the rate of neural differentiation [9, 10]. To clarify whether irisin plays an important role in neurogenic regulation, Moon and colleagues showed that irisin increases cell proliferation in mouse H19-7 HN cells via STAT3, but not AMPK and/or ERK, whereas irisin has no dose-dependent effect on neurite outgrowth and synaptogenesis in these cells [47] (Table 1). Together, these results demonstrate that irisin mediates neuroprotective effects partly through activation of the STAT3 signaling pathway. Through a combination of its neuroprotective property and its induced neural differentiation through MAPK signaling pathway, irisin likely serves important function to support neuronal health.

### Fndc5 attenuates inflammation and insulin resistance via AMPK pathway

Obesity is a complex disease that triggers inflammation and macrophage accumulation in adipose tissue and subsequently leads to metabolic diseases, including type 2 diabetes and insulin resistance [48–50]. Recent studies have shown that improved hyperlipidemia and increased lipolysis are two consequence of *Fndc5* overexpression in adipose tissues of obese mice [51]. Guo-Qing Zhu’s research group demonstrated that *Fndc5* deficiency reduced insulin sensitivity in obese mice. Their findings showed that Fndc5 plays a critical role in attenuating adipose tissue inflammation and insulin resistance [51]. The improvement effects of Fndc5 are significantly alleviated by the AMPK inhibitor Compound C (CC), but did not change by utilizing an AMPK activator, AICAR. These data demonstrate that AMPK reduces inflammation and M1 macrophage polarization by Fndc5 [52] (Table 1). This function of irisin, compared with those described already for irisin through MAPK signaling, demonstrated that irisin exerts its effects through alternative pathways.

### Irisin exerts its anti-metastatic effects via the PI3K/AKT pathway in lung cancer tissue

One of the pivotal pathways in cancer cell growth, proliferation, and survival, is the PI3K/AKT pathway which is elevated in a variety of cancers including ovarian, breast and pancreatic cancers [53–55]. Irisin inhibits the migration, proliferation, and invasion of lung cancer cells and reduced the expression of EMT markers by inhibiting the PI3K/AKT pathway. From a mechanistic perspective, irisin can reverse the activity of epithelial–mesenchymal transition (EMT) and inhibits the expression of Snail via the PI3K/AKT pathway [56]. Specifically, irisin inhibited EMT and reduced the invasion of lung cancer cells via the PI3K/AKT/Snail pathway [56]. Conversely however, increased irisin levels may have protective roles in liver cancer cells through partial activation of the PI3K/AKT pathway, which may facilitate liver cancer progression and decrease the sensitivity to chemotherapy [57] (Table 1).

### Effect of irisin on migration and invasion of osteosarcoma cells through the STAT3/Snail signaling pathway

EMT is a cellular process which occurs during normal embryonic development and wound healing. It is a highly conserved process, by which epithelial cells lose their cell polarity and cell–cell adhesion [58]. IL-6 appears to promote the proliferation, metastasis and angiogenesis of osteosarcoma through several downstream signals including AKT, ERK1/2 MAPK and STAT3 [59–61]. Kong and colleagues showed that irisin treatment of osteosarcoma cells inhibited the proliferation, migration and invasion of osteosarcoma cells by reversing IL-6-induced EMT. In a further study, it was ascertained that irisin inhibits IL-6-induced STAT3 phosphorylation [62] (Table 1).

### Irisin suppresses pancreatic cancer cell growth via the activation of AMPK

AMPK-mTOR is a major signaling pathway in progress of pancreatic cancer. Irisin administration reportedly suppresses pancreatic cancer cell growth via the activation of AMPK and downregulation of the mTOR pathways, thereby inhibiting EMT of pancreatic cancer cells [63]. Moreover, irisin is responsible for increasing caspase activity in a process called “attenuation of cell death resistance”. Notably, irisin also suppresses other hallmarks of cancer such as “maintaining proliferative signaling” through targeting the PI3K/Akt pathway and also “evading growth suppressors” through the AMPK-mTOR pathway. In summary, irisin exerts cancer suppression through reduction in proinflammatory cytokines and adipokines linked to obesity status.

### Browning of WAT in myostatin-knockout mouse through activating AMPK-PGC1α-FNDC5 signaling pathway

Myostatin, a myokine released by myocytes, acts on muscle cells by inhibition of myogenesis, muscle cell growth and differentiation. *Myostatin* (*Mstn*) knockout mouse showed significantly more muscle mass [64]. It has been shown that WAT of *Mstn*^−/−^ mice show attributes of BAT through significantly increased expression of BAT marker genes, including *Pgc1α* and *Ucp1* [65]. Moreover, in muscles of *Mstn*^−*/*−^ mice, the level of total AMPK and activated pAMPK increased significantly as AMPK-PGC1α-Fndc5 pathway was activated in the muscle of *Mstn*^−*/*−^ mice, leading to increased production of irisin [65] (Table 1).

### Anti-inflammatory properties of irisin are connected to TLR4/MyD88 signaling pathway activation

Irisin also acts as an adipokine and exerts a potential protective effect on the progress of obesity-related diseases, such as arteriosclerosis, insulin resistance, and type 2 diabetes. Potential anti-inflammatory properties of irisin have been demonstrated [66]. Macrophage RAW 264.7 cells stimulation with lipopolysaccharide (LPS; 100 ng/mL) and irisin pretreatment caused a dramatic decrease in Toll like receptor (TLR4) and Myeloid differentiation primary response protein 88 (MyD88) levels, and decreased the phosphorylation of nuclear factor-κB (NF-κB), thereby reducing the release of vital pro-inflammatory cytokines (IL-6, TNF-α, and IL-1β) and keratinocyte chemo attractant (KC) and monocyte chemotactic protein 1 (MCP-1). Moreover, irisin exerts this anti-inflammatory effect through phosphorylation of MAPKs, where a significant reduction in p-JNK and p-ERK but not p-p38 was observed [66]. In conclusion, potential protective effects of irisin against the development of diseases associated with obesity, may be attributed in part to irisin’s anti-inflammatory properties (Table 1).

### Negative regulation of serum irisin and skeletal muscle Fndc5 by SMAD3 during exercise

Smad3^−/−^ mice transform WAT to BAT-like cell phenotype, thereby inferring protection against high fat diet-induced obesity and type 2 diabetes mellitus [67]. Irisin induces WAT browning similar to that observed in SMAD3-deficient mice [3]. SMAD3 represses *Fndc5* and *Pgc1α* expression in skeletal muscle and in Smad3^−/−^ mice exercise increases serum irisin and skeletal muscle Fndc5 as well as its upstream activator Pgc1α to a greater extent than in wild-type mice [67] (Table 1).

### Irisin lowers blood pressure via the AMPK-Akt-eNOS-NO pathway in endothelial cells

Hypertension, affecting approximately one billion people worldwide, is a major risk factor for a variety of diseases including coronary artery disease, stroke, heart failure, atrial fibrillation, peripheral vascular disease, vision loss, chronic kidney disease, and dementia [68]. Exercise is a nonpharmacological anti-hypertensive factor responsible for lowering blood pressure through unknown mechanisms. Due to the close relationships between metabolic diseases and hypertension, it is postulated that exercise may act through irisin to elicit lowering of blood pressure [68]. AMPK could be activated by irisin, thereby down regulating intracellular ATP levels through increasing reactive oxygen species (ROS) or intracellular calcium concentrations [35]. In the vasculature, activated endothelial AMPK phosphorylates eNOS, stimulating NO release and subsequent vasodilation of both large conduit and resistance arteries [68]. This was demonstrated in the spontaneously hypertensive rat, where irisin lowers blood pressure by ameliorating endothelial dysfunction of the mesenteric artery through the AMPK-Akt-eNOS-NO signaling pathway [68]. In a similar study, irisin improved endothelial function in aortas of high fat diet-induced obese mice through activation of AMPK-eNOS signaling [69] (Table 1).

### Antidepressant-like effect mediated by Fndc5/BDNF/Akt in mice by modulating hippocampal signaling pathway

There is mounting evidence for significant involvement of creatine in the pathophysiology of major depressive disorder (MDD) [70]. Several studies have shown that Akt and its downstream molecular targets effect MDD and may be targets for depression treatment [71–73]. It has shown that the acute antidepressant-like effect of creatine is dependent, at least in part, on PI3K/Akt signaling pathway activation [74]. AKT induces activation of BDNF [75, 76]. BDNF is a member of the neurotrophin family of growth factors, which regulates the survival and growth of neurons and has recently received attention in relation to the therapeutic action of antidepressant treatment [77, 78]. Several studies demonstrated that physical exercise increases BDNF levels in the hippocampus through PGC-1α activation and *FNDC*-*5* expression modulation [44, 79, 80]. Subchronic administration of creatine in the hippocampus caused increased expression of *PGC*-*1α, FNDC5* and *BDNF* through the FNDC5/BDNF/Akt pathway [81] (Table 1).

### Irisin improves cardiac hypertrophy by inducing protective autophagy via mTOR independent activation of AMPK-ULK1 and AMPK- mTOR pathways

FNDC5 overexpression attenuated damage to transverse aortic constriction induced hypertrophy in the heart, demonstrating a protective effect of irisin against cardiomyocyte hypertrophy induced by angiotensin II or phenylephrine. Irisin deficiency decreased autophagy, whereas irisin overexpression elevated autophagic flux. ULK1 plays an essential role in the initiation of autophagy and can be regulated by AMPK and mTOR via direct phosphorylation at Ser555 and Ser757, respectively. Irisin increased the activity of AMPK but not Akt and MAPK in hypertrophic hearts and cultured cardiomyocytes which triggered further activation of ULK1 at Ser555 but not Ser757 and did not affect the mTOR-S6K axis [82]. Irisin may also display anti-fibrotic therapeutic potential to counter angiotensin II-related cardiac fibrosis. In skeletal muscle cells the ADAM family of metalloendopeptidases, especially ADAM10, is responsible for the cleavage of FNDC5 into irisin and for irisin-induced cardiac autophagy through activation of the AMPK-mTOR pathway. Since FNDC5 expression is significantly decreased in ischemic cardiomyopathy, in severe chronic heart failure mice, application of irisin may be beneficial as a novel therapeutic approach for treatment of heart disease [83] (Table 1).

In addition, FNDC5/irisin is responsible for the repair of cardiac tissue after an ischemic heart disease episode as it induces cell proliferation through activation of cardiac progenitor cells. In this context, there is a significant increase in proliferative markers such as Ki67 and phosphorylated histone 3, a reduction of histone deacetylase 4, and increased p38 acetylation in Irisin-treated cardiac progenitor cells [84].

## Discussion

Irisin is a myokine that is secreted from skeletal muscle in response to exercise and stimulates convertion of WAT to BAT [3]. There is rising evidence to support a vital role for irisin in the regulation of metabolism and body fat reduction [85]. These effects can raise energy expenditure, increase oxygen consumption and reduce insulinemia [86].

However, more recent studies revealed additional vital roles for Fndc5/irisin in other tissues [36, 87, 88]. Recent studies have revealed that Irisin has anti-cancer, -depression, -hypertension and -cardiac hypertrophy properties [57, 62, 68, 81–83].

The inhibitory effects of irisin on inflammation is mediated by significant decrease in the release of vital pro-inflammatory cytokines. This property is associated with hyperphosphorylation of MAPKs induced by irisin [66]. Therefore, irisin acts as an important regulator of tissue cross-talk, mainly between muscle and other tissues/organs. Therefore, irisin is a potential option for preventing/treating a wide range of diseases including cancer [89]. In the present review we tried to delineate molecular mechanism of intracellular functions of irisin. However, more investigations are needed to clarify the precise mechanism of irisin actions and its spectrum of physiological effects.

## Conclusion

Numerous studies demonstrate the physiological properties of irisin, pointing to its beneficial health potential in the maintenance of a variety of tissues and organs. With the promise of its health benefits, further studies to investigate and test the therapeutic applications of this signaling peptide are highly anticipated. Understanding the precise underlying mechanisms of Fndc5 is required to fully appreciate and appropriately apply Fndc5/irisin in cancer, aging and other metabolic diseases.

## Data Availability

Not applicable.

## Abbreviations

AD: Alzheimer’s disease
Ang II: Angiotensin II
ATGL: Adipose triglyceride lipase
BAT: Brown adipose tissue
BDNF: Brain-derived neurotrophic factor
BFP: Body fat percentage
CC: Compound C
EBs: Embryoid bodies
ERK1/2: Extracellular signal-regulated kinase 1 and 2
EMT: Epithelial–mesenchymal transition
FAS: Fatty acid synthase
FNDC5: Fibronectin type III domain-containing 5 protein
FRCP2: Fibronectin type III repeat containing protein
JNK: c-Jun N-terminal kinases
HUVEC: Human umbilical vein endothelial cell
IL-1β: Interleukin 1β
IL-6: Interleukin 6
KC: Keratinocyte chemoattractant
LPS: Lipopolysaccharide
MAPKs: Mitogen-activated protein kinases
MAPKAPKs: MAPK activated protein kinases
MAPKK: Mitogen-activated protein kinase kinase
MAPKKK: Mitogen-activated protein kinase kinase kinase
MCP-1: Monocyte chemotactic protein 1
MDD: Major depressive disorder
MyD88: Myeloid differentiation primary response protein 88
mESCs: Mouse embryonic stem cells
NF-κB: Nuclear factor-κB
NLK: Nemo-like kinase
P-ERK: Phosphorylated ERK
PE: Phenylephrine
Pep: Peroxisomal protein
PGC-1α: Peroxisome proliferator-activated receptor-γ coactivator-1 alpha
PKA: Protein kinase A
P-p38: Phosphorylated p38
PPAR-γ: Peroxisome proliferator-activated receptor-γ
RA: Retinoic acid
RAR: Retinoic acid receptor
RARE: Retinoic acid response element
ROS: Reactive oxygen species
SAPK: Stress activated protein kinases
TAC: Transverse aortic constriction
T2DM: Type 2 diabetes mellitus
TNFα: Tumor necrosis factor α
UCP: Uncoupling protein
WAT: White adipose tissue
